# Long-term, repeated doses of intravenous autologous mesenchymal stem cells for a patient with Parkinson's disease: a case report

**DOI:** 10.3389/fneur.2023.1257080

**Published:** 2023-09-27

**Authors:** Ridhima Vij, Alan Prossin, Mallika Tripathy, Hosu Kim, Hyeonggeun Park, Thanh Cheng, Djamchid Lotfi, Donna Chang

**Affiliations:** ^1^Hope Biosciences Research Foundation, Sugar Land, TX, United States; ^2^McGovern Medical School at UTHealth Houston, TX, United States; ^3^Hope Biosciences, Sugar Land, TX, United States

**Keywords:** mesenchymal stem cells, adipose derived, autologous, Parkinson's disease, case report

## Abstract

Parkinson's disease (PD) is a neurodegenerative disease that involves the loss of dopaminergic neurons in the substantia nigra pars compacta of the basal ganglia. Clinically, patient presentation involves a combination of motor and non-motor symptoms characterized by progressive worsening over time and significant decreases in overall quality-of-life. Despite there being no fully restorative cure for PD, Mesenchymal Stem Cell (MSC) therapy offers promising therapeutic potential. Here, we report a case of a 77-year-old female, living with idiopathic Parkinson's Disease for over 17 years. The patient received multiple infusions of autologous Hope Biosciences adipose-derived MSCs (HB-adMSCs). A total of 26 infusion treatments of HB-adMSCs were administered over the course of ~2 years that resulted in marked improvements in her typical Parkinsonian symptoms, as demonstrated by the decreases in her UPDRS (Unified Parkinson's Disease Rating Scale) scores. Changes in clinical scores mirrored concurrent changes in regional brain metabolism as quantified by FDG-PET (Fluorodeoxyglucose-Positron Emission Tomography), compared to baseline. Long-term, multiple infusions of HB-adMSCs were safely tolerated by the patient without any serious adverse events. Further research is needed to evaluate the safety and efficacy of HB-adMSC therapy for the treatment of PD patients.

## 1. Introduction

Parkinson's Disease (PD) is a progressive neurological disorder characterized by the preferential loss of dopaminergic neurons in substantia nigra with involvement of multiple other cell types throughout the central and peripheral nervous system. PD is the second most common neurodegenerative disease after Alzheimer's disease that affects 2–3% of the population ≥65 years (1, 2). Pathogenesis of PD involves the progressive dopaminergic neuron degeneration, which leads to the loss of stimulation of the direct motor pathway and the loss of inhibition of the indirect motor pathway of the basal ganglia (3, 4).

Currently, there is no cure for PD, but patients are often candidates for medication-based treatment and, in some cases, neurosurgical intervention like deep brain stimulation (DBS) (5, 6). Among the numerous available medications non-ergot dopamine agonists as well as levodopa/carbidopa combination are usually first-line treatments. However, with levodopa-carbidopa therapy, there is an “on-off” phenomenon that occurs with progressive disease, which results in improved mobility during the “on” periods, but impaired motor function during the “off” periods (7).

Because of limited effectiveness, and the side effects associated with many of the available PD medications, alternative therapeutics are constantly under study. Owing to their anti-apoptotic, immunomodulatory, paracrine, and multidirectional differentiation abilities, mesenchymal stem cells (MSCs) are currently being evaluated in clinical trials to treat neurological disorders (8). Specifically, MSCs secrete neurotrophic factors, such as glial cell line-derived neurotrophic factor and nerve growth factor, which prevent the dopaminergic neurons from undergoing apoptosis and promote neurogenesis (9). Preclinical studies using bone marrow-derived MSC therapy have also demonstrated prevention of degeneration of dopamine-producing neurons (10). However, other studies have reported contradictory effects of MSC transplantation on PD treatment, demanding the need for further research (11, 12).

In this case report, we sought to demonstrate tolerability and efficacy of multiple infusions of HB-adMSCs in improving the signs, symptoms, and overall quality-of-life for a woman with Parkinson's Disease.

## 2. Case presentation

### 2.1. Case history

Herein, we present a case of a 77-year-old, white, non-Hispanic or Latino female diagnosed with PD in 2004, suffering from severe neurodegenerative deterioration for over 17 years. The patient's weight, height, and BMI at screening were 57.5 kg, 157.5 cm and 23.2 kg/m^2^ respectively. What started out as a left-hand tremor progressed to cogwheel rigidity, bradykinesia, chorea, athetosis, dystonia, truncal swaying, head tremor and dyskinesia. Her past medical history displayed several other nervous system and musculoskeletal disorders that included: Kyphosis, Tendinitis de Quervain's, Distal Neuropathy, Restless Leg Syndrome, and insomnia, along with a history of fractures in her rib and LS spine. Her surgical history included total hip replacement, tonsillectomy, and cosmetic eye surgery. She experienced a combination of side effects and resistance to treatments, some of which included dyskinesia associated with Comtan, nausea and vomiting with Azilect, and the on-off phenomenon linked to Sinemet (which the patient started taking in 2013). The patient's complete medication regimen included: Sinemet (25/100 mg) 5x daily, Rytary (36.25/145 mg) 2 capsules 4x daily, Azilect (1 mg) daily, Comtan (200 mg) 5x daily, Neupro (4 mg) transdermal daily, Requip (8 mg) daily, Ambien (10 mg) nightly, Melatonin (15 mg) nightly, Gabapentin (300 mg) prn, and Tylenol (1,000 mg) prn. Despite being on all these medications, it was only enough to help her walk; she still experienced frequent falls, severe dyskinesias, and repetitive episodes of freezing. She would slide out of the chairs she sat in, was unable to turn around in bed, had very stooped posture, and required the assistance of an in-house caregiver. There were not many restorative treatment options available for this patient after she exhausted the extensive list of failed medications.

**Figure 1:**
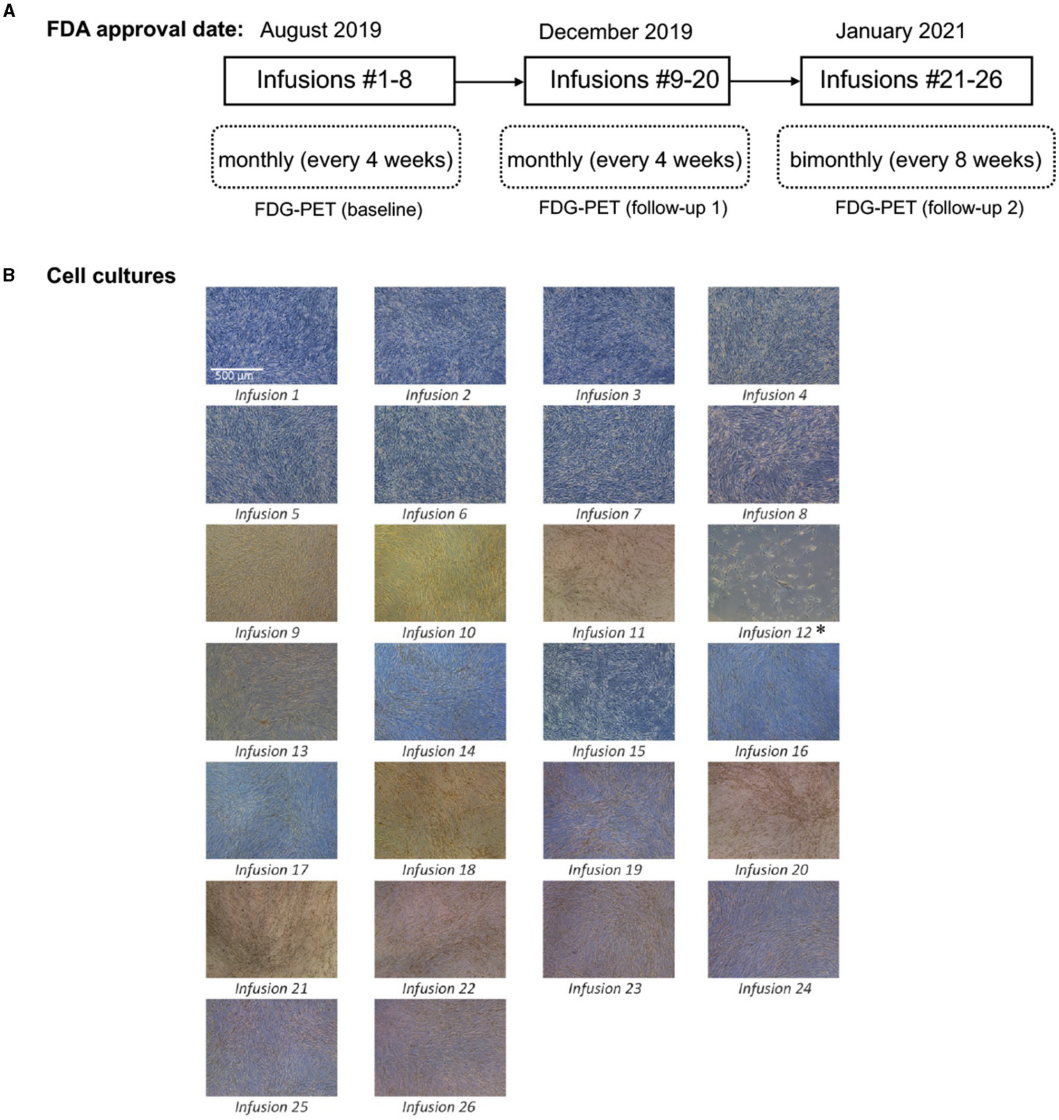
**(A)** Protocol approval timeline; **(B)** Cell culturing images for 26 infusions. Passage 4 culture images for all 26 infusions. Images were taken with a Leica inverted microscope at 50x magnification. Color variation is due to flask wall thickness, angle and light. *The product released for infusion #12 was lower than the minimum dose requirement of 200 (±20%) million live cells. Low cell count was because of compromised cell growth due to growth medium issue.

In August 2019, FDA authorized an expanded access protocol for intravenous administration of eight infusions of autologous HB-adMSC treatment for this PD patient. The patient responded quite well to the therapy with no safety concerns that led us to continue her treatment, and in December 2019, FDA approved another set of 12 monthly infusions. The infusion treatments resulted in positive findings associated with significant improvements in patient's parkinsonian symptoms. After completing 20 infusion treatments, FDA approved 6 more infusions to the patient's protocol in January 2021, which continued at the same dose, but the interval changed from every 4 weeks to every 8 weeks (Figure 1A). A total of 26 infusions were received by the patient over the course of ~2.5 years.

### 2.2. Isolation of patient's adipose derived MSCs

To isolate autologous adMSCs, 10 ccs of adipose tissue was extracted via liposuction, from the patient's abdomen. The extract was then tested by the quality control unit at Hope Biosciences LLC., for USP71 sterility and mycoplasma due to possible contamination from the fat extraction procedures, followed by centrifugation to phase-separate the adipose tissue. A total of 5 mL adipose tissue was then treated with collagenase to isolate stromal vascular fraction (SVF). Cells from the SVF were plated in Hope Biosciences' HB-103 medium to establish a P0 culture. The resulting adherent cells were further cultured with HB-101, Hope Biosciences' growth medium (with 5% FBS) and the MSCs were cryopreserved in Hope Biosciences' proprietary freezing medium (containing 10% DMSO) at passages #0, #1 and #2 to create a complete cell bank for the patient. An aliquot of #2 culture supernatant was cleared by the quality unit for USP71 sterility, mycoplasma, and endotoxin. For infusions, passage #2 cells were thawed, recovered in passage #3, and cultured to passage #4 (Figure 1B). Final release of the purified harvest of passage #4 cells was cleared by the quality unit for final release for USP71 sterility, mycoplasma, and endotoxin, gram stain and cell characterization via flow cytometry performed on a ThermoFisher AttuneNXT flow cytometer using labeled antibodies. Operation of the flow cytometer consists of using single color control samples to adjust the corresponding PMT voltage and gain. Then, an unstained cell sample is used to adjust the forward and side scatter gain, and to set the gate on the negative populations across all channels of interest. Single color controls are used to calculate the compensation matrix, which is then applied to the experiment and used to create an analysis template. This template is saved and used to analyze the final product cell samples using the Attune Software. Once completed, data analysis tables are created to summarize the results. The immunophenotype of all cultured HB-adMSCs are evaluated by QC to confirm the identity and purity as well as ensure that cells remain undifferentiated prior to release. A total of 26 infusions (manufactured from the cell bank created for the patient and freshly harvested from passage #4; Figure 1B), each with 200 million ±20% MSCs mixed in 20 mL of 0.9% sterile sodium chloride were administered intravenously over a period of ~2.5 year: 20 monthly infusions and remaining 6 infusions administered bimonthly. Each lot passed cGMP compliant quality control standard assessments to ensure a standardized product is delivered for each treatment and was administered within 48 h of packaging. Quality assessments included viability; appearance; sterility (USP71); gram staining; mycoplasma; endotoxin; and cell identity/purity as indicated by MSC defining surface markers (CD73+, CD29+, CD31- and CD45-) (Table 1).

**Table 1:** Infusion details for all 26 infusions with MSC quality control metrics.

**Infusion #**	**Date of administration (month/day/year)**	**Total cell count (million)**	**Cell viability (%)**	**CD73 (%)**	**CD29 (%)**	**CD31 (%)**	**CD45 (%)**
1	08/22/2019	168	98.13	99.28	99.97	0.28	0.08
2	09/05/2019	232	97.32	95.66	99.95	0.18	0.09
3	10/17/2019	165	98.10	98.39	99.89	0.31	0.08
4	11/14/2019	238	97.39	98.92	99.93	0.40	0.18
5	12/12/2019	229	97.9	98.51	99.96	0.55	0.38
6	01/09/2020	226	98.60	96.19	99.89	0.59	0.19
7	02/06/2020	170	97.25	98.46	99.99	0.37	0.17
8	03/05/2020	192	97.56	99.17	99.82	0.31	0.07
9	04/02/2020	230	97.30	99.83	99.97	0.44	0.10
10	04/30/2020	192	93.02	99.10	99.90	2.88	0.60
11	05/28/2020	195	89.71	97.26	99.94	1.52	0.34
12	06/25/2020	27.2^*^	94.44	87.78	96.56	2.72	0.91
13	07/23/2020	234	98.65	95.58	99.54	0.15	0.31
14	08/20/2020	160	96.15	97.24	98.6	0.07	0.07
15	09/17/2020	239	93.00	87.71	98.49	0.06	0.13
16	10/15/2020	186	93.55	86.63	98.33	0.28	0.28
17	11/12/2020	176	92.44	97.58	98.33	0.00	0.00
18	12/10/2020	230	96.00	95.76	98.73	0.06	0.18
19	01/07/2021	168	94.59	94.81	99.37	0.19	0.00
20	02/04/2021	244	98.51	95.12	99.14	0.00	0.10
21	04/01/2021	218	94.44	86.76	96.29	2.59	0.40
22	05/26/2021	176	93.22	95.21	99.37	0.00	0.13
23	07/20/2021	208	97.01	99.24	99.83	0.00	0.17
24	09/16/2021	125^**^	100.0	96.52	99.54	0.00	0.23
25	11/10/2021	240	95.35	97.49	99.91	0.00	0.38
26	01/05/2022	202	96.92	98.77	98.77	0.00	0.29

The product released for infusion #12 and #24 was lower than the minimum dose requirement (200 million live cells ± 20%).

* Low cell-count because of compromised cell growth due to growth medium issue.

** Low cell-count due to low cell-yield from the patient. All HB-adMSCs were positive for CD73 and CD29 and negative for CD45 and CD31 cell surface markers, as expected.

### 2.3. Treatment and results

After receiving ~ first 10 infusions, noticeable improvements were seen in her posture, accompanied by less frequent dyskinesias, or freezing, and no tremors. Additionally, she exhibited a normal gait and an overall improvement in her ability to carry out activities of daily living (ADLs) independently. These improvements were notable given the severity of her symptoms before receiving the intervention. Before MSC infusions, she was on very high doses of a variety of medications and required a 24-h live-in caregiver. However, following the HB-adMSC intervention, we observed notable improvements in her PD symptoms with greatly improved quality-of-life. Moreover, she discontinued her Ambien after experiencing significant improvements following 12 infusions and her neurologist also discontinued Sinemet, Azilect and Comtan along with 25% reduction in Rytary. She also started to prepare meals and perform other household chores and no longer required the help of a caregiver. To assess the effectiveness of HB-adMSC therapy, several evaluation methods including UPDRS scores, neuro-quality-of-life assessments, and neuroimmune imaging with FDG-PET, were employed.

#### 2.3.1. Unified Parkinson's disease rating score

**Figure 2:**
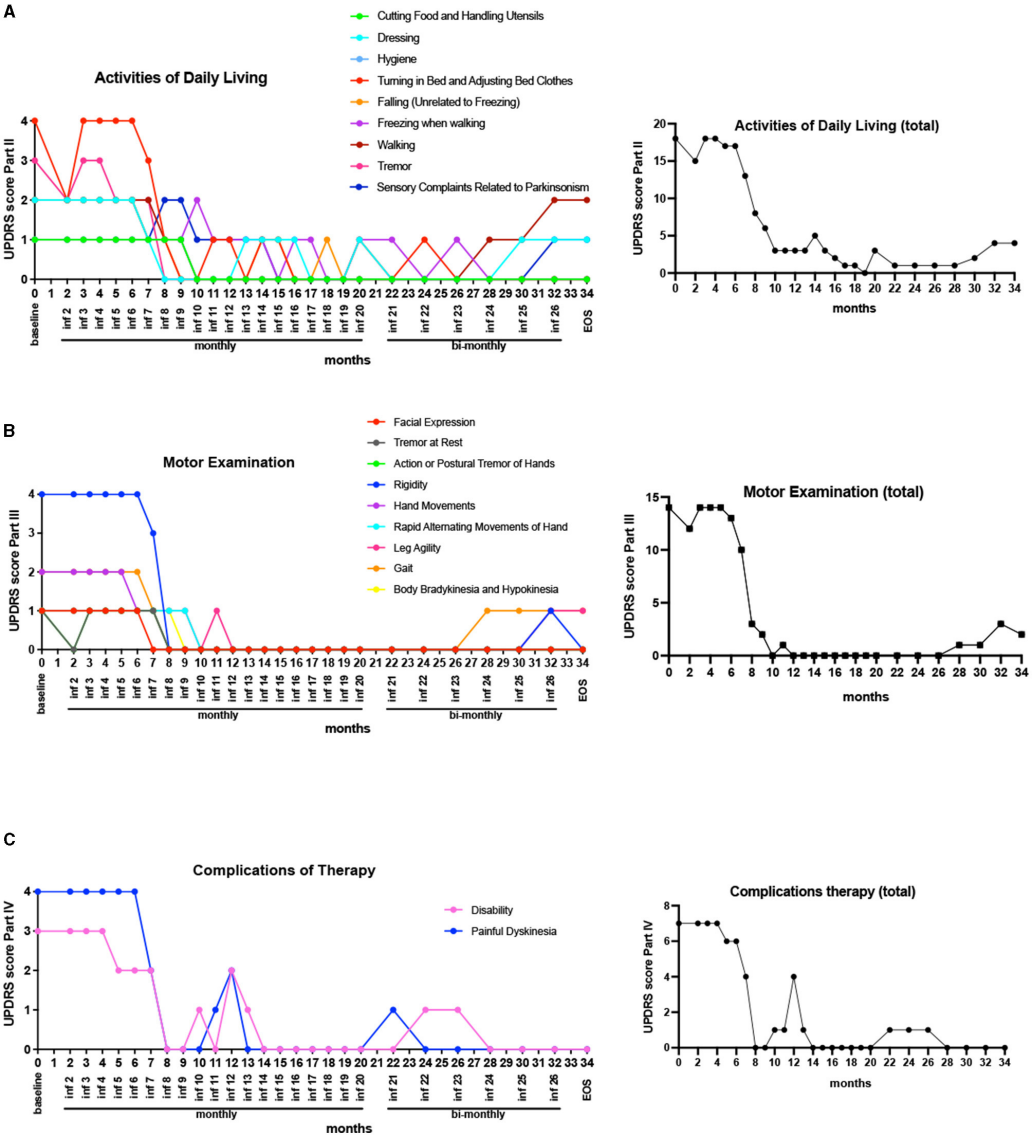
UPDRS scores **(A)** Activities of Daily Living (part II): Improvements were seen in all measured parameters with significant improvements observed in “turning in bed and adjusting bed clothes” as well as “tremor” (left), with overall improvement in the total score (right); **(B)** Motor Examination (part III): Major improvements were noted in “rigidity” after only eight infusions of HB-adMSC therapy. Other motor symptoms that showed improvements included “facial expression,” “tremors at rest,” “hand movements,” “leg agility,” “gait,” and “body bradykinesia and hypokinesia” (left), with substantial improvements in the total score (right); **(C)** Complications of Therapy (part IV): Significant improvements were observed in patient's “painful dyskinesia” (left) with an overall improvement observed in the total score (right).

UPDRS assessment tool was used to evaluate motor functionality of the patient. Specifically, the parameters assessed under the UPDRS evaluations were (1) Activities of Daily Living (UPDRS part II), (2) Motor Examination (UPDRS part III), and (3) Complications of Therapy (UPDRS part IV). The patient showed significant improvements in each of these areas—post-therapy, the patient demonstrated remarkable improvements in UPDRS part II, for each of the activities of daily living, compared to baseline (Figure 2A, left). Also, the total score for UPDRS part II showed significant improvements (score improved from 18 to 4) (Figure 2A, right). Similarly, several improvements were seen in the areas of motor examination UPDRS part III scores, with the most notable changes observed in patient's rigidity (Figure 2B, left). There was an overall improvement in her motor symptoms that included, tremors at rest, hand movements, leg agility, gait, as well as body Bradykinesia and Hypokinesia (the total UPDRS part III score improved from 14 to 2) (Figure 2B). Additionally, following HB-adMSC therapy, the patient's disabling dyskinesias were no longer severe; the total UPDRS IV score improved from 7 to 0 (Figure 2C).

#### 2.3.2. Imaging: fluorodeoxyglucose-positron emission tomography scan

A Parkinson's Disease Related Pattern (PDRP) has been known to be identified with FDG-PET, outlining brain regions of hypometabolism (parieto-occipital and pre-motor cortices) and other regions of normal or hyper-metabolism (cerebellum, pons, thalamus, and lentiform nucleus), associated with disease severity, progression, and treatment response (13–15). For this patient, three FDG-PET scans were completed to differentiate metabolically active brain regions from less metabolically active regions in a PDRP type pattern. A baseline FDG-PET scan was obtained on August 20^th^, 2019, a comparison FDG-PET scan (follow-up 1: FU1) obtained post-adMSC intervention, on August 11^th^, 2020, and a final FDG-PET scan (follow-up 2: FU2) was obtained on March 24, 2022. The first two scans were completed on a GE Discovery LS PET/CT scanner (GE Medical Systems) at the PET Imaging of Houston facility (Houston, TX) where CT attenuation correction was applied. The final scan was completed on a Siemens Biograph scanner (Siemens Healthcare GMBH) at the HMI-Richmond facility (Houston, TX) where CT attenuation was applied. Imaging analyses were completed in PMOD software wherein the individual SUV (standardized uptake value) images were calculated, smoothed (3 mm Gaussian kernel), co-registered, and normalized to the MNI template (Montreal Neurological Institute, Montreal, CA). Resulting SUV images were subsequently normalized to their respective whole brain average SUV by simple division to yield final SUVR images for comparison. In this case report, using the baseline image, we identified regions with relatively low metabolism within the parieto-occipital and those with relatively high metabolism within the cerebellum and thalamus, consistent with PDRP conceptualizations in PD. We then compared SUVR values in those regions, at the baseline to FU1 and FU2, to determine whether the pattern of changes in metabolism in these regions were consistent with patterns of clinical changes.

**Figure 3:**
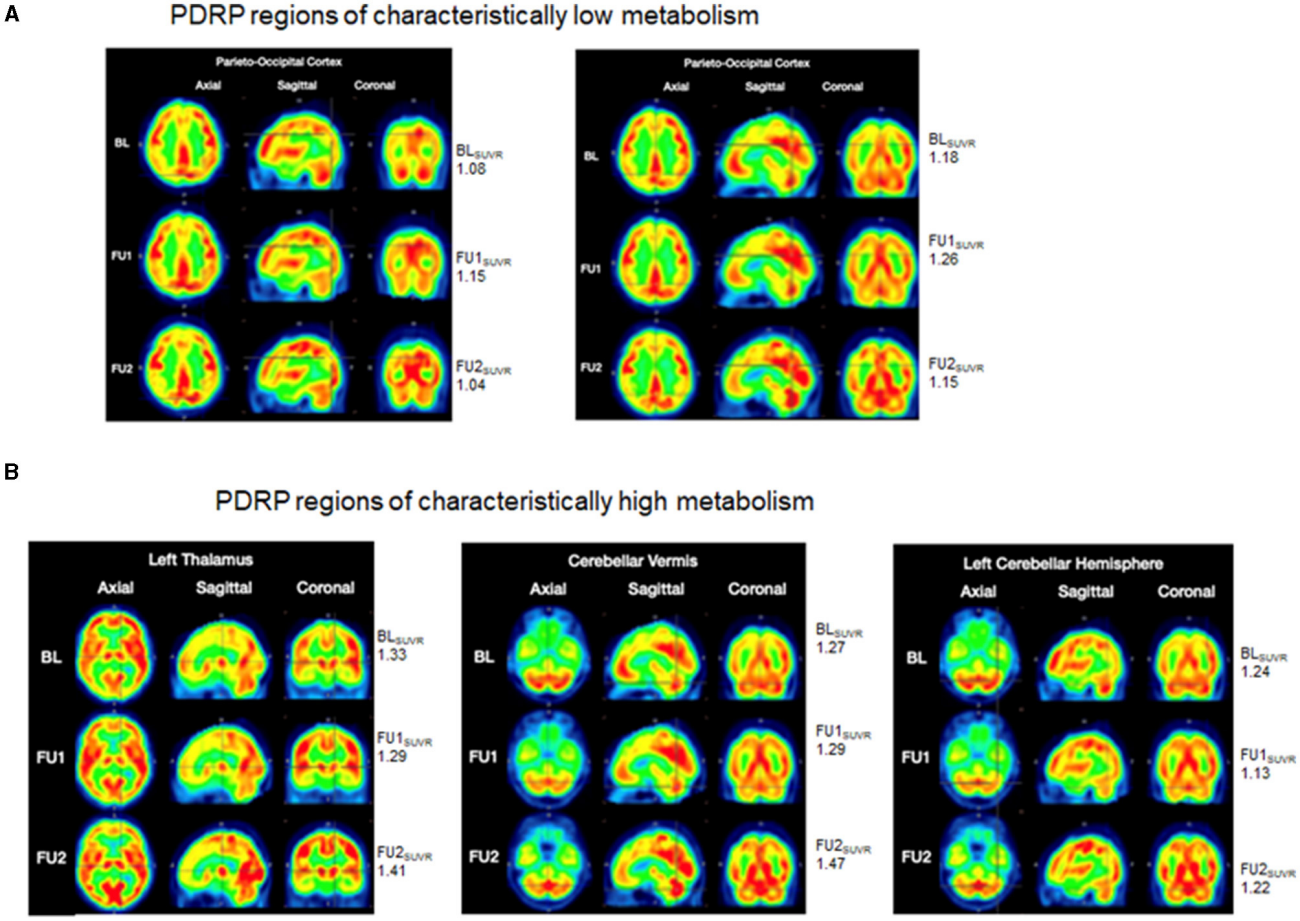
FDG-PET scans. Brain metabolism is represented via a color spectrum with SUVR values ranging from low to high, consistent with colors ranging from blue/green/yellow (low SUVR) to orange/red (moderate SUVR) and bright red (high SUVR). **(A)** PDRP regions of characteristically low metabolism (parieto-occipital cortices): (left) cross-hairs focus on a region with relatively low metabolism at BL (SUVR = 1.08) that is increased at FU1 (SUVR = 1.15) and exhibits a subsequent decline at FU2 (SUVR = 1.04); (right) cross-hairs focus on the another portion of the parieto-occipital cortex with relatively lower metabolism at BL (SUVR = 1.18) that is increased at FU1 (SUVR = 1.26) and subsequently declines at FU2 (SUVR = 1.15); **(B)** PDRP regions of characteristically high metabolism images (thalamus, cerebellar cortices): (left) cross-hairs are centered on the left thalamus wherein relatively higher metabolism at BL (SUVR = 1.33) was slightly reduced at FU1 (SUVR = 1.29) and subsequently increased at FU2 (SUVR = 1.41); (middle) cross-hairs center on the cerebellar vermis with minimal change in SUVR values from BL (SUVR = 1.27) to FU1 (SUVR = 1.29) and subsequent increase to FU2 (SUVR = 1.47); (right) left cerebellar hemispheres show a reduction in SUVR values from BL (SUVR = 1.24) to FU1 (SUVR = 1.13) and a subsequent increase to FU2 (SUVR = 1.22). BL, baseline; FU1, follow-up 1; FU2, follow-up 2.

In the parieto-occipital cortex, relatively low metabolism was seen at the baseline (SUVR = 1.08), that was increased at FU1 (SUVR = 1.15) with a subsequent decline at FU2 (SUVR = 1.04) Figure 3A (left). The cross-hairs in Figure 3A (right), centered on a portion of the parieto-occipital cortex, showed relatively lower metabolism at baseline (SUVR = 1.18) that got increased at FU1 (SUVR = 1.26) with a subsequent decline at FU2 (SUVR = 1.15). As demonstrated in Figure 3B (left), SUVR values reduced from 1.33 at baseline to 1.29 at FU1 and subsequently increased to 1.41 at FU2. Similar findings were observed in the right thalamus. The cross-hairs in Figure 3B (middle), centered on the cerebellar vermis showed minimal change in SUVR values from baseline (SUVR = 1.27) to FU1 (SUVR = 1.29) with subsequent increase at FU2 (SUVR = 1.47). Similarly, cross-hairs (centered on an aspect of the left cerebellar hemispheres) in Figure 3B (right) where a reduction in SUVR values from baseline (SUVR = 1.24) to FU1 (SUVR = 1.13) and subsequent increase at FU2 (SUVR = 1.22) was noted. Similar changes were observed in various locations throughout the left cerebellar hemisphere and in the right cerebellar hemisphere.

#### 2.3.3. Neurology Quality-of-Life assessments

Neuro-QoL survey was used to assess multidimensional aspects that include physical, mental, as well as social wellbeing. Improvements were noted in several areas of the assessment. For example, there were mild improvements in patient's cognitive function and her satisfaction with social roles and activities along with keeping up her social commitments. There were also improvements in her ability to keep up with work responsibilities, with improvements in her anxiety raw score (improved from 23 to 19, post-therapy), along with improvements in her feelings of exhaustion. Additionally, the assessment for upper and lower extremity function also demonstrated slight improvements in the score (correlated with UPDRS part II and III). However, several other parameters in the assessment remained stable with no signs of improvement or worsening.

#### 2.3.4. Laboratory evaluations

Standard laboratory measures of complete blood count (CBC) and comprehensive metabolic panel (CMP) and coagulation panel (CP) were performed at various timepoints to evaluate the safety of the treatment. The laboratory values did not show any unusual changes when compared to baseline. Also, no treatment-related adverse or any serious adverse events were reported.

## 3. Discussion

Currently, there are no fully restorative treatments available for PD. The controversial long-term effects of the available therapeutics for PD paves way for MSC therapy. Mechanistically, MSCs secrete neurotrophic factors, thereby possess the potential to prevent apoptosis of dopaminergic neurons and stimulate neurogenesis through the release of fibroblast growth factor 2, vascular endothelial growth factor, endothelial growth factor, and more (16–18). There is also evidence to suggest that MSCs may play a role in limiting the degeneration of dopaminergic neurons (9).

Previous studies have demonstrated the efficacy of MSCs in PD patients. Boika et al. (19) used bone-marrow derived MSCs, to assess changes in motor and non-motor symptoms, using various scaled assessments and showed improvements in PD symptoms. Additionally, other studies employed stereotactic administration of bone-marrow derived MSCs into the sublateral ventricular zone in patients with PD (20, 21). Although improvements in PD symptoms were reported in these studies but only a fraction of patients demonstrated persistent improvements.

In this case report, administration of multiple infusions of HB-adMSCs resulted in substantial improvements in the patient's PD symptoms as demonstrated by the UPRDS scores that also corelated with the improvements seen on the FDG-PET scans. Consistent with the results of this report, another study by Shigematsu et al. also demonstrated improvements in the clinical scores (UPDRS) using multiple infusions of adipose-derived MSCs in three PD patients (22). However, the improvements in the clinical score results were not evaluated further with any imaging or any other clinical assessments. For this patient, in addition to numerous physical improvements, she also experienced several positive changes in measures related to her quality-of-life. It should, however, be noted that the improvements were more pronounced and remained stable up to infusion #20, with a slight worsening in some of the UPDRS scores around infusion #21–26. These clinical findings also correlated with the final FDG-PET scan where reduction in regions of hypometabolism and enhancements in regions of hypermetabolism were noted, compared to scan at FU1. This phenomenon could be attributed to the change in the dosing interval from monthly to bimonthly (infusion #1–20 were administered every 4 weeks while infusions #21–26 were administered every 8 weeks). A similar trend was observed in one of our previous case report where the relapse of symptoms was observed in a patient with Systemic Lupus Erythematosus (SLE) when the dosing regimen was changed from every 4 weeks to every 8 weeks (23). Taken together, the results of this study demonstrate the need for optimal treatment frequency to overcome persistent degeneration associated with such diseases, however, further research with larger sample size is needed to confirm these findings.

The patient's neuro quality-of-life assessments did not correlate well with the improvements seen in the UPDRS scores and showed limited to no improvement at all when compared to the baseline. A possible explanation for these results may be attributable to the differences in the way these assessments are made—neuro-QoL are self-based assessments compared to the UPDRS scoring criteria that is purely based on the clinician's ratings—thus, better reflects the treatment outcome. Given the limited availability of data from a single patient with idiopathic PD, a larger sample population might be necessary to understand a correlation between neuro-QoL assessments with other PD evaluations.

Also, we would like to acknowledge potential limitations related to the FDG-PET imaging analyses. The values reported are in SUVR units, reflecting the ratio of regional SUV over the SUV averaged over the whole brain. While this method can be useful when attempting to normalize data obtained across different scanners (e.g., FU2 scan was obtained from a different scanner than the baseline scan and FU2 scan), this scanner harmonization method is not perfect and can potentially lead to misleading results.

## 4. Conclusions

Overall, HB-adMSC therapy was efficacious in improving the patient's experience with a progressively degenerative neurological disease such as PD. Administration of monthly HB-adMSCs infusions had a promising therapeutic effect, specifically at the symptomatic levels of PD. Post-therapy, the patient experienced less dyskinesias, had pronounced improvements in her tremors, and had regained a significant level of independence. These results were in stark comparison to her experience while on the medications, during which she needed help with most ADLs. Also, HB-adMSCs therapy was well-tolerated by the patient. Given the progressive, chronic nature of Parkinson's disease, additional research using HB-adMSCs should be conducted to confirm the findings of this study.

## Data availability statement

The original contributions presented in the study are included in the article/supplementary material, further inquiries can be directed to the corresponding author.

## Ethics statement

The studies involving human participants were reviewed and approved by Western Institutional Review Board, Inc. Washington, USA. The patients/participants provided their written informed consent to participate in this study. Written informed consent was obtained from the patient for the publication of any potentially identifiable images or data included in this article.

## Author contributions

RV: Formal analysis, Visualization, Writing—original draft, Writing—review and editing. AP: Formal analysis, Visualization, Writing—original draft, Writing—review and editing. MT: Writing—original draft. HK: Resources, Writing—original draft. HP: Resources, Methodology, Writing—review and editing. TC: Data curation, Investigation, Supervision, Writing—review and editing. DL: Data curation, Investigation, Supervision, Writing—review and editing. DC: Conceptualization, Funding acquisition, Project administration, Writing—review and editing.
